# Localisation and cross-border assistance to deliver humanitarian health services in North-West Syria: a qualitative inquiry for *The Lancet*-AUB Commission on Syria

**DOI:** 10.1186/s13031-019-0207-z

**Published:** 2019-05-27

**Authors:** Diane Duclos, Abdulkarim Ekzayez, Fatima Ghaddar, Francesco Checchi, Karl Blanchet

**Affiliations:** 1 Health in Humanitarian Crises Centre, London School of Hygiene and Tropical Medicine, London, UK; 2 Research for Health in Conflict in Middle East and North Africa (R4CH), King’s College London, London, UK; 3 Department of Epidemiology and Population Health, Faculty of Health Sciences, American University of Beirut, Beirut, Lebanon

**Keywords:** Humanitarian aid, Cross-border assistance, Syria, Global health, Local actors

## Abstract

**Background:**

In a growing number of humanitarian crises, “remote management” is negotiated across borders and implemented by humanitarian agencies through “local actors” to deliver assistance. However, the narrative describing the involvement of local actors in the delivery of humanitarian aid in armed conflict settings remains reductionist and unreflective of the complex and circular course of the “localisation of aid”. This paper explores cross-border humanitarian assistance within the Syrian conflict. We document how humanitarian actors operate to deliver humanitarian health care in North-West Syria (Turkish border), explore their challenges and critique the language used within current debates on the localisation of aid.

**Methods:**

We undertook key informant interviews with Turkey-based humanitarian aid professionals involved in the humanitarian health response inside Syria. We integrated data previously collected for *The Lancet*-American University of Beirut Commission on Syria during field work in Gaziantep, Turkey, through meetings, conversations, discussions and expert consultations with Syrian health professionals, WHO-Turkey staff members and members of Syrian health directorates. We also drew from background desk reviews conducted by the Commission on health systems responses and timeline of events in Turkey during the Syrian conflict.

**Results:**

This paper uncovers creative and effective bottom-up strategies that enhanced cross-border coordination of aid delivery into Syria. Our findings unravel the key role played by Syrian providers in accessing vulnerable populations and in reshaping coordination and funding mechanisms inside Syria, as well as the disproportionate risks local actors bear within the response. Our findings also reveal an iterative negotiation of decision-making dynamics, a “low-profile approach” promoted to gain access to populations of concerns, and an environment that is heavily shaped by close interpersonal relationships and social trust.

**Conclusions:**

Our multifaceted narrative unpacks circular flows of interactions among actors and uncovers strategies developed by practitioners on the field, which are often left undocumented. We argue that there is an opportunity for the humanitarian sector to learn from these synergies to rethink how medical humanitarianism is framed (hopefully leading to a more collaborative framing that resists mainstreaming “local” actors within a “traditional” system). There is also an opportunity for the humanitarian and global health communities to reflect on how value attributed to human lives needs to be questioned in contexts where national staff face a disproportionate risk to deliver aid.

## Background

In armed conflict settings, international humanitarian agencies generally adapt their structures and operations in response to constraints in securing humanitarian space. Increasingly, working through various local aid actors with better access to affected populations is preferred to direct operations. In a growing number of humanitarian crises, “remote management” has been negotiated across borders and implemented by humanitarian agencies to deliver humanitarian assistance, often with a local actor implementing services on the ground [1]. Narratives of this modality of delivering aid arguably remain reductionist, particularly with regards to the involvement of local actors and the underlying political nature of the localisation of aid. Language used to refer to the roles and integration of local actors into the humanitarian system (“localisation”) has nonetheless been evolving, as exemplified by the 2016 World Humanitarian Summit. Notably, the Summit’s Grand Bargain agreement commits “donors and aid organizations to provide 25 per cent of global humanitarian funding to local and national responders by 2020” through “multi-year investment in the institutional capacities of local and national responders […] and [incorporating] capacity strengthening in partnership agreements”. [2, 3] These statements acknowledge a need to reconsider power relationships within the humanitarian landscape and that reinforcing the role of local actors necessitates building their capacities. As for the political nature of the localisation of aid, it has also been increasingly recognised in humanitarian conversations, contrasting with views considering localisation as a logistic exercise [4].

Cross-border humanitarian practices and remote management have been used in the past in the humanitarian sector, as exemplified in conflict settings such as Somalia, South Sudan, Iraq and Afghanistan. This history of humanitarianism, has shaped the ways in which humanitarian agencies have responded to the conflict in Syria:

“[One] direct response to rising insecurity has been remote management, wherein intervening aid organizations have responded to heightened risk by withdrawing key senior international staff and upper national management from the conflict zone, and instead relying on local staff or partners to continue programming at reduced levels. The second is an industry trend towards the localization or local ownership of programs” [5].

The logic supporting the localisation agenda is multi-layered. On one operational level, “local actors” are de facto players who are already driving responses during natural disasters (e.g. the 2004 Indian Ocean tsunami), epidemics (e.g. 2014 West Africa Ebola) and conflict settings such as Syria where international actors may lack secure access [6]. On another operational level, it is recognised that only a less bureaucratic, decentralised humanitarian architecture could lead to more effective responses, and that local actors with their sustained presence also provide a bridge from humanitarian assistance to development. [7] At the governance level, localisation challenges a certain ontology of power governing the western-led humanitarian system in which partnerships are forged in such ways that international western actors hold power over local partners.

The main critique to localisation of aid to date comes from humanitarian actors expressing concerns about the capacity of local actors to scale up interventions, and about the political neutrality of local actors in times of conflicts. The former argument may hold true in many instances and can be partially addressed with adequate training and capacity building, while the latter tackles the humanitarian principles of “neutrality”, and needs some broader political reflection within a context of violence. Localisation discussions to date overlook that being labelled as “local” comes with a number of risks, including jeopardizing one’s life to care for others. The very term “local actors” remains loose, referring to “a very wide range of different stakeholders: from government authorities at various levels, via NGOs, other civil society groupings and private businesses, to every native individual or community [members]” [7].

Given the above, documenting the role played by and the experiences of emergent local actors in humanitarian responses, particularly the Syrian context, has become of interest to us. The rationale behind this is that unravelling the experiences of local actors — including their coping mechanisms as well as the burden and trauma they bear in the process — and integrating them within humanitarian discussions and coordination mechanisms could contribute to imagining a more equitable and efficient aid delivery model; one that would not end up reproducing the very power relationships that it sets out to address.

The Syrian conflict provides an important case study with respect to the localisation of aid and the language used to unpack it. While the Syrian government has long stopped supporting medical facilities in opposition territories [8], access to conflict-affected populations by humanitarian agencies inside Syria has been challenging since the start of the conflict. The lack of guaranteed security for staff, complicated by constant threats and attacks on health care workers and facilities [9], have led most international organisations (IOs) and International Non-Governmental Organisations (INGOs) to operate remotely across the Turkish, Jordanian, Lebanese and Iraqi borders through local partnerships [10], while other IOs and INGOs have chosen to manage their humanitarian operations from Damascus, Syria. In that respect, the role of “local actors” has become prominent and essential. Still, “Syrian actors’ share of international humanitarian funding in no way matches their role in the actual delivery of assistance” [11].

We present a case study (North-West Syria – Turkish border; September 2017) of the above dynamics, while relying on information gathered earlier in 2017 (May and July 2017) as well as multiple desk reviews. The analysis is presented through a multifaceted narrative that unpacks the circular flows of interactions and dynamics among actors, which, in turn, contributes to the development of a dynamic framing of medical humanitarianism. In contrast to the classic model of international health response, we draw on Hilhorst and Janssen’s work on the everyday politics of aid [12], to explore how the language of medical humanitarianism is being (re)articulated and (re)implemented in different ways by different sets of health actors in highly political and unstable spaces. We also draw on the concept of therapeutic geographies used to “account for the remapping of health care” in conflict [13]. Our empirical perspective offers logistical insights into the cross-border practices of healthcare services provision inside Syria and contributes to the discussions on the institutionalisation of a “politics of life” [14], whereby Syrian first responders and health providers are pushed to accept a disproportionate safety risk to ensure service implementation.

## Methods

### Study setting

At the time of our study in September 2017, access to the 70-some functioning health facilities in North-West Syria was still constrained by major challenges of safety and security, financing and coordination. Bombings were intensifying in North-West Syria affecting hospitals run or supported by the organisations in which some of our informants worked — these organisations had to operate with minimum staff or close. Country directors and field coordinators were communicating with their staff on Skype, WhatsApp, as well as with ground informants providing regular security updates. International humanitarian staff based in Turkey had generally not been able to visit projects inside Syria since late 2013. However, Syrian staff regularly crossed into Syria, apart from when border crossings were insecure. Hence, NGOs relied extensively on their Syrian cross-border staff and on the internet to communicate with their partners inside Syria. Information was flowing between Syrian medical doctors from inside and outside Syria, Gaziantep-based NGOs and the UN agencies to gather information to anticipate attacks on or near health facilities, and make informed decisions on whether facilities should be running with minimal staff or closed altogether. Communication and decision-making mechanisms seemed to reflect well-established practices as interviewees constantly reflected on the problems they faced and the solutions they identified during earlier similar episodes. The simultaneous proximity and tangible distance between actors in Turkey and in Syria were amplified by the immediate danger faced by staff on the ground in Syria.

Cross-border relationships between humanitarian actors in Gaziantep and Syria are rooted in historical and former socio-economic ties, which are constantly reshaped by local and national politics in Turkey. During the last century of Ottoman rule in the Levant, for instance, Gaziantep (then known as Antep) was administratively attached to the Aleppo vilayet [15]. Around 80 years later in 2005, Aleppo and Gaziantep became official “twin cities”, “an initiative taken by local authorities to bolster economic, social and cultural ties between its peoples” [2]. Flows of people and goods between both cities intensified after that and “despite a sharp decrease of 45% in 2012, the following period from 2010 to 2015 saw a drastic increase of 237% owing largely to the local firms’ share in delivering humanitarian assistance across the border” [2]. In 2014, resolution 2165 adopted by the Security Council allowed the United Nations (UN) and its partners to deliver cross-border humanitarian assistance to Syria. In 2015, the UN Office for the Coordination of Humanitarian Affairs (OCHA) established one of its regional hubs for cross-border humanitarian assistance in Gaziantep. The Turkish Red Crescent played a key role in facilitating the logistics of the cross-border operations. Though collaborations between national and international humanitarian actors had started in 2012, the 2014 resolution was a milestone to institutionalise those links. Within this cross-border humanitarian landscape, Turkey played a key role in enabling Syrian national NGOs to facilitate cross border referrals. However, international and within-Turkey politics have affected cross-border activities. As of January 2015, “the introduction of new regulations [had] adversely affected [the ability of] Syrian staff from NGOs […] providing assistance to Syria […] to cross the border regularly to accompany shipments and conduct programming activities” [16]. Furthermore, in March 2016, the Turkey-EU “refugee deal” was signed, allowing Greece to return migrants irregularly crossing the Aegean Sea to Turkey in exchange for visa facilitations financial support for Turkey’s refugees’ population. Since 2017, restrictions and bureaucratic delays to deal with INGO proposals and actions imposed by the Turkish government and lack of coordination between government institutions have posed mounting challenges to ongoing cross-border work. After March 2017, Turkish authorities requested both INGOS and Syrian NGOs re-register and re-apply for staff work permits, some of which were then rejected. According to the press [17], these bureaucratic steps mainly targeted large Western organisations.

### Data sources

Our analysis relied on multiple data sources. We undertook a desk review to identify programmatic documents on cross-border activities implemented in North-West Syria. These included OCHA humanitarian bulletins reporting on cross-border activities from Turkey into Syria as well as health cluster bulletins that reported on the humanitarian response in Syria more broadly (June 2015–September 2017) [18]. In September 2017, we undertook ten key informant interviews in the Turkish border city of Gaziantep with humanitarian aid professionals (Syrian NGOs personnel’s, Syrian clinicians, UN and INGOs staff) involved in the health humanitarian response inside Syria. A pre-agreed topic guide informed our interviews, with a certain degree of flexibility to adjust to unanticipated themes emerging from our informants’ narratives. In addition, we drew on field notes from the Research and Documentation Team (RDT) at *The Lancet*-AUB Commission on Syria (hereafter referred to as “the Commission”) gathered during fieldwork in Turkey in May and July 2017 – this included notes from meetings, conversations, discussions and expert consultations with Syrian health professionals, WHO-Turkey and members of Syrian health directorates (health directorates related to the interim Ministry of Public Health [8], emerged in opposition areas in Syria to coordinate health activities) [19]. We also drew on background desk reviews undertaken by the RDT – this included summaries from the grey literature on health systems responses and timeline of events in Turkey during the Syrian conflict.

### Analysis

We used a thematic qualitative analysis. We reviewed the key themes that had been identified before data collection from our desk reviews and literature searches, and identified unanticipated themes that emerged from the interviews. These themes were iteratively discussed within the research team to locate participants’ views and experiences of cross-border interventions in the humanitarian literature on the localisation of aid. For the scope of this paper, we adopted terms such as “international NGOs”, “national” “NGOs”, “local actors” and “diasporas groups” as used by policy documents reviewed and interviewees, and merely reflected on their political implications and validity from the perspective of the lived experience of providing health services inside Syria.

### Ethics

Ethics approval was granted for this study by the London School of Hygiene &Tropical Medicine. Meetings and consultations done by the American University of Beirut were considered by the Institutional Review Board as part of the general work of the Commission. For the key informant interviews, written or oral informed consent was sought from participants and confidentiality and anonymity of our informants was ensured. Interviews were audio recorded unless interviewees expressed preference otherwise, in which case detailed notes were taken by researchers. Interviews that were recorded and notes were stored in a secured password protected hard drive, and accessed only by the co-authors who conducted the interviews. For the fieldwork undertook by the RDT, interviewers from the Commission obtained oral clearance from interviewees to take notes. Notes were stored securely and were anonymised and aggregated to protect the identities of the interviewees and synthesize information.

## Results

Interactions among a wide range of humanitarian actors to deliver health services inside Syria from Turkey offer original insights into the politics and logistics of such partnerships. On the one hand, efforts have been made to integrate Syrian NGOs in the international response through the formalisation of coordination structures (humanitarian coordination meetings initially, and later a Humanitarian Liaison Group) and pooled funding mechanisms as well as the activation of UN-led clusters [20]. Syrian NGOs have also opted for a more formal structure by creating multiple coordination platforms over the years — such as the Syrian NGO alliance or the Syria Relief Network — to facilitate engagement between Syrian and international humanitarian actors. In addition, we will show that national NGOs also bring into these structures local medical staffs from inside Syria with whom they partner to deliver services to individuals and communities.

### The “low-profile” and “close-ties” approach to humanitarian access in northern Syria

Beyond the narrow “humanitarian access” terminology (which may reduce humanitarian routes to logistic considerations), lies a key challenge in creating or developing effective inter-organisational and interpersonal relationships among non-state actors collaborating to deliver an intervention in areas where parties in the conflict directly target health providers. Humanitarian access in Syria has been constrained by several issues that have evolved over time, including conflict and attacks on access routes, restrictions imposed by the Syrian government and parties to the conflict and targeting of healthcare workers and facilities. Access challenges have created the essential conditions for IOs and INGOs to work in partnership with national NGOs and medical actors in Syria to reach the most vulnerable, using a low-profile approach: “the use of smaller tonnage and fewer vehicles at a time, with no branding, and the use of commercial carriers are common modalities employed” [21].

In addition to this low-profile approach, our interviews with Syrian doctors working in the Idlib province in Syria revealed the centrality of interpersonal trust to enable any collaboration with Syrian and non-Syrian actors offering support from outside the country in the early phases of the conflict; a phenomenon reminiscent of the literature citing the importance of social trust in improving cooperative relations [22, 23]. In the context we studied, trust was often present with networks previously forged, or when being introduced by a trusted intermediary. For example, while starting to establish networks with expatriate doctors from outside Syria, doctors from Idlib initiated limited communication with few focal points, using pseudonyms, relying on Virtual Private Networks (VPNs) to hide their internet access, using Subscriber Identity Modules (SIM cards) with unknown accounts, etc. Networks were also mobilised inside the country to channel medical supplies across military obstacles, e.g. through doctors sometimes relying on women from their family, as they were less likely to be searched at checkpoints. Within this context, “frontline health workers” become “undercover relief workers”, putting personal relationships at the centre of professional partnerships. Even though the humanitarian and medical work was institutionalised through local NGOs and INGOs over time, interpersonal relationships were key to facilitate the development of a coordinated health response. While interpersonal connections were crucial in such a context, it emerged from some accounts that they can also burden responders, as personal disagreements could sometimes affect professional relationships.

### Funding landscape mechanisms

Funding sources for the humanitarian and medical cross-border response in North West Syria were diverse. Some of the main ones were institutional donors such as governments or the European Commission Humanitarian Fund (ECHO), which were largely covering the majority of the humanitarian cross-border operations in North-West Syria. The main channel for this type of funding was through IOs or INGOs, which were either carrying out the implementation themselves or subcontracting Syrian NGOs. Funding also came from philanthropists. This type of funding was channelled through either INGOs, Syrian NGOs or even grassroots organisations that had no registration in neither Damascus nor neighbouring countries. However, this funding was running short as the crisis prolonged. Diaspora networks, composed of Syrian expatriates who established networks in their countries of residence, also supported the humanitarian response in Syria. The early diaspora networks were mainly medical ones. Some of these networks were institutionalised and converted into NGOs such as the Syrian American Medical Society (SAMS) and the Syrian Expatriates Medical Association (SEMA). Since both NGOs had wide networks of members and supporters, they had flexible funding through membership fees and private donations. This made them more independent from donors’ money fluctuations and constraints. For example, their private fund (20–30% of the overall funding) was used in besieged areas where donors were reluctant to fund projects. In addition, this flexible fund allowed these NGOs to fill gaps caused by withdrawal of some INGOs after financial cuts by donors. For instance, SEMA’s flexible funds allowed it not to withdraw from any project it had started in the past and fill the gaps caused by the withdrawal of INGOs. At the time of our interviews, this latter phenomenon was happening - key international humanitarian actors were withdrawing from health facilities and SEMA was among the NGOs to cover these gaps alongside with SAMS and the Union for Medical and Relief Organisations (UOSSM).

Since 2014 and following United Nations Security Council Resolutions 2139 and 2165, Syrian NGOs have had direct access to international funds through the Humanitarian Pooled Fund (HPF), a multi-donor, earmarked fund managed by UNOCHA to fund the Humanitarian Response Plan. All international and national NGOs registered in UNOCHA coordination platforms were eligible to apply for this fund, but this required registration in Turkey (or another country) and UNOCHA validation of the organisation’s capacity to manage resources. Registered NGOs were then classified into three levels of liability, which determined not only their eligibility but also the funding ceiling. This adaptation of a global funding scheme to the localisation framework in Syria was key to facilitate direct funding from UNOCHA to avoid duplication of effort.

Our research identified several challenges for funding cross-border activities, including the logistics for cash flow inside Syria. The collapse of the banking system in opposition-controlled areas in North-West Syria, for example, and the absence of registered money transfer agencies left humanitarian actors with no option but to rely on local money transfer agencies (sometimes referred to as *Hawalah*). NGOs rely on these agencies to transfer funds from NGO offices and bank accounts in Turkey to NGO staff or partners in Syria. Another main challenge was related to the ambiguity of the Turkish government legislations in relation to the use of the above-mentioned system, as there was no previous legalisation in place in relation to such system for money transfer to cover cross-border activities. While the Turkish government was trying to develop a legalisation to balance between the urgent needs to transfer money for the operations inside Syria and the need to fully control the money movement across the border, most cases related to this issue were discussed through unofficial channels between NGOs and the Turkish government.

### A bottom-up approach to coordination

Complex, bottom-up, personal relationship-driven channels of coordination emerged among responders implementing medical interventions in areas outside the control of the regime. These ultimately shaped global coordination mechanisms for health inside opposition-controlled areas. “Trust” and “intersubjectivity”, which also manifested themselves in the “low profile” and “close ties” approach to humanitarian access discussed above, were central to these coordination channels, and can only be captured outside a restrictive emergency framework.

“From the beginning, we were trying to organise ourselves (…). Sometimes Idlib had no needs so they send to Homs, trying to organise the response. We deal with the doctors from outside individually at the beginning and they know us with our fake names. Inside, there are two kinds of doctors: field doctors and management doctors who deal with logistics and coordination. Not everyone knows the structure of the group for security issues.” (Syrian clinician).

Inter-individual relations and social networks were key to develop connections between doctors outside and inside Syria as this was a context where health providers were imprisoned and where the long-term presence of secret intelligence in the country affected trust among citizens. Expatriate doctors divided responsibilities for different regions in Syria according to the access they had through professional and family networks and contacts. Technicians and experts in telecommunication from outside and inside the country were involved as well and provided VPN accounts for activities.

The role played by Syrian providers to coordinate health activities inside Syria has evolved throughout the conflict. Syrian health professionals started to organize inside Syria forming medical committees and health directorates. This progressively expanded outside the Syrian borders as Syrian NGOs began to have a more active stance in international coordination mechanisms and as coordination agencies began to recognize entities inside Syria (such as health directorates). The role of the multiple interim governments of opposition in the cross-border activities in North-West Syria was limited because of issues related to legitimacy, stability and resources. In general, it took until 2015 for the coordination to evolve on both sides of the border including both national and international actors:

“At the beginning we suffered (…) in 2013 when the health working group started in Antakya. The Syrian NGOs were seated in a corner without any contribution. It was not respectful. All the international staff would speak, and Syrians would stay quiet. One woman who worked with an international NGO supported us to be on the table with the other players…Now the health cluster speaks Arabic and there is a co-lead from a Syrian NGO. Coordination between Syrian NGOs improved, and we advocated that our priority be the priority of the health cluster.” (Syrian clinician, Turkey-based Syrian NGO staff member).

Such examples are key to understand the realities of building up coordination mechanisms in a transnational humanitarian context while working at the intersection between health networks that had never met before the crises. They show that coordination cannot always be channelled down through vertical processes. While reflecting on her/his practice during the interview, one of our informants, who was country director for an INGO, emphasized the value of coordination mechanisms initiated by Syrian doctors. Such value lay in the efficiency of those networks in maintaining communication between different areas and quickly mobilising resources and response to emergencies. Over the years, these mechanisms became integrated into more formal platforms of coordination such as the health cluster in Gaziantep. However, this bottom-up approach is still the backbone of the coordination mechanisms.

### Cross-border monitoring and evaluation

In a context where organisations work remotely to deliver medical aid inside Syria, third party monitoring has been used by some organisations. [24] Monitoring and evaluation companies were established in Turkey with field staff and networks inside Syria. These companies are contracted by INGOs or Syrian NGOs to monitor and evaluate their projects inside Syria. Some donors and INGOs made this third party monitoring a reporting requirement for any project conducted remotely in Syria. However, some of these monitoring mechanisms have become challenging in light of reports of private monitoring firms disrupting relationships between NGOs and beneficiaries on the ground as well as relationships between NGOs and donors [24]. Humanitarian organisations thus developed creative ways to monitor the implementation of their intervention without physical access to the field and to report to their funders. For instance, following the Office of U.S. Foreign Disaster Assistance (OFDA) investigation that happened in Turkey in 2016 [25], some INGOs implemented new monitoring tools such as photographic evidence, GPS location evidence, and paper work (e.g. receipts).

International humanitarian staff who, before working on the Syrian crisis, used to work in coordination positions for their organisations, acknowledge that coordinating a mission always involved working remotely, typically being based in the capital, with monthly field visits. In contrast, humanitarian field logisticians felt that their role was redefined by the lack of access to the field, and part of their professional identity was challenged. In Turkey, one of the organisations we visited had lost its registration, making it impossible for staff based on each side of the border to cross for over a year. The absence of a direct relationship between IOs staff and beneficiaries restricted efforts to improve accountability to populations in need. In some cases, community health workers recruited by organisations we approached were considered as being the “voice of the community” inside Syria. The pathways by which community health workers can truly channel beneficiaries’ views and concerns need to be better understood. However, there were some innovative approaches to engage local communities in the process of remote monitoring through triangle agreements between INGOs, implementing Syrian NGOs and local councils or local committees. Threeway-communication channels were set up in these agreements to ensure quality and compliance. Still, other barriers were faced by humanitarian organisations to measure the performance of their cross-border programmes in the Syrian context. To start with, there was limited circulation of documents, either because that could put local partners and organisations at risk by rendering their action too visible or because there was lack of trust in the effectiveness and neutrality of some of the coordination mechanisms that were led either by UN agencies or INGOs that had presence in regime-controlled areas. This sometimes led to a lack of transparency in reporting mechanisms [24]. The lack of coordination and standardization processes on monitoring and evaluation mechanisms among funders was an additional constraint faced by local partners in Syria [24].

### Localisation processes beyond emergency response

Partnerships developed over time to deliver or support the provision of health services inside Syria have made visible the dynamic character of medical humanitarianism. In this section, we will draw on some of our findings on the cross-border response to reflect on longer-term implications for the Syrian health system and for the humanitarian sector.

Any health interventions in a humanitarian context can lead to innovations in the health system, and in Syria, funding channelled through cross-border mechanisms shaped how health services were prioritised by the local health authorities formed in opposition-held areas. [26]. The implementation of an intervention to build capacity among midwives through training existing midwives and creating a 3-year programme for new recruits is a good example of such synergies between humanitarian interventions and local health programmes. According to one of our study participants involved in this initiative, the idea of this intervention was born from a simple observation: there were fewer gynaecologists in Syria, yet a large pool of trained midwives persisted; however, Syrian midwives were not working up to international guidelines. During the needs assessment phase of the intervention, discussions took place between the reproductive healthworking group in Gaziantep and providers in Syria to identify which resources were needed at each level of the health system, and what steps should be taken to have a more effective system. The Minimum Initial Service Package (MISP) was used as an evidence-based approach, and it was compared to the existing health system structure in Syria, with the aim of decentralising reproductive health services. Community health workers were tasked with providing advice and identifying women at risk of pregnancy complications so that they could be referred. The overall strategy took nine months of negotiations with local health workers: doctors, in particular, needed to be convinced that midwives could gain the required skills to work independently and provide good standards of care. Midwives were identified during trainings to act as trainers themselves in Syria. Schools of midwifery were opened, and agreements were made to issue diplomas that would be delivered by the organisation, health directorates and the opposition ministry of higher education.

### Humanitarian principles in tension

Humanitarian principles (humanity, impartiality, neutrality and independence) were well known by all international and Syrian NGOs working on the cross-border response. However, the implementation and monitoring of these principles through cross-border health activities was challenging, especially in a context of remote training of health providers and remote monitoring of activities. Some relief workers we interviewed raised specific concerns around the implementation of neutrality. Local medical and humanitarian workers have their own political views, they argued, particularly after witnessing crimes committed by parties to the conflict and being threatened or persecuted by the same parties. Therefore, it might be challenging to be neutral. However, the same workers had no issues with being impartial. For example, one interviewee shared an incident where a soldier of the Syrian army was brought to a hospital supported by a Syrian NGO. He was provided with the required medical care before opposition groups took him. At the same time, this NGO and the hospital staff identified themselves as part of the revolution against the Syrian government. This account by no mean suggests that humanitarian actors’ neutrality and impartiality in a conflict — including local actors —can be taken for granted. However, such accounts question the problematic assumption that local actors are by essence less prone to be neutral and impartial.

In this study, it was important to reflect about values and principles emerging outside the centres of humanitarian action. In our interviews with Syrian doctors involved in the humanitarian response in Syria, for example, “localisation” emerged as a key principle that needs to be unpacked. Localisation was valued by local NGOs providing health services, given their understanding and proximity to the context and the people affected by the crisis: “We are the sons of this country”, stated one of the Syrian doctors we interviewed in Gaziantep to justify the focus his organisation puts on never interrupting the provision of health services. In our informants’ discourses, this was associated with a focus on continuity of health service provision, as opposed to international organisations.

Localisation was also perceived as a means to avoid “trends” in interventions that were not adapted to the field and that might not be sustainable:

“We don’t believe in mobile clinics as a long-term solution. Equipment, labs, privacy of the doctors. At a certain point in time, mobile clinics became like a fashion trend. The health system before the war did not have any mobile clinics. It is hard for doctors to go to the areas with the mobile clinics. It is exhausting. They already work in several facilities. In some areas, it was useful to detect malnutrition and refer them to hospitals. One organisation wants to implement 25 mobile clinics and wants to put all the resources in these clinics. We used to have mobile operation rooms, but it was dangerous for doctors. Everything regarding the health project is dangerous and mobile clinics are not the solution; even if the car is not visible, the crowd will be visible” (interview with Syrian doctor).

Although we cannot offer a comprehensive picture accounting for the pros and cons of facilities like mobile clinics, these field-based perspectives shed light on how humanitarian interventions were framed by Syrian humanitarian responders, as a reality check on innovation brought (and sometimes imposed) by international partners and funders.

### Study limitations

The spectrum of informants we interviewed for this study is limited and does not reflect the full range of humanitarian actors involved in the cross-border assistance for health services from Turkey to North-West Syria. Notably we did not interview Turkish actors involved in the response. In addition, the statements of informants operating inside Syria were exclusively collected from Turkey as we did not conduct fieldwork in North-West Syria. A comprehensive political economy analysis is also lacking in our analysis, which could have played a key role in determining the attitude, motivations and some practices of local actors in North-West Syria. However, such analysis was beyond the scope of this study.

## Discussion

While we acknowledge the dynamism of cross-border partnerships in general, both the partnerships and health interventions developed to provide aid in North-West Syria challenge the typical “humanitarian architecture model” and uncover the adaptability of complex humanitarian systems. There is also growing evidence accounting for convergences between international and local systems in Syria that go beyond a strict division between system and non-system actors [20].

In the context of increased international acknowledgement that the humanitarian landscape needs to include local actors driving responses to crises, the health response inside opposition-controlled areas in North-West Syria sheds light on the social networks that have developed across borders to support or operate health services, their transformation into more formal coordination structures (such as the formation of Syrian NGOs alliance, the Syria Relief Network, inter-cluster coordination mechanisms, coordination with local health entities, etc.…), and the challenges encountered (such as changes in the Turkish border management and Turkish government relationship with INGOs). Intense circular flows of persons, information and norms across health networks to deliver medical aid inside Syria suggest that it is “time to let go” of strict categorisation and hierarchy of actors [27]. Our findings do not support the validity of a model in which local partners can be “remotely managed” or “encouraged” from outside Syria [1]. Dewachi and colleagues used the concept of therapeutic geographies “defined as the geographic reorganisation of health care within and across borders under conditions of war” [13]. Beyond providing access to populations, these changing therapeutic geographies are challenging the architecture of humanitarian assistance. Only if we acknowledge that cross-border interventions do not only reflect a shift in the management of human resources in humanitarian assistance to provide access, but an in-depth change in implicit rules governing decisions in different sites of the humanitarian system, can we improve accountability mechanisms to populations included or excluded from these dynamic systems, and, *in fine*, imagine the future of humanitarian interventions. This requires engaging with the political dimension of healthcare in conflict settings where the lines between warfare and healthcare are increasingly “blurred” [28]. In North-West Syria, caring for injured demonstrators or fighters, or building alternative forms of governance of health services provision, is seen as a challenge to State authority. This renders health providers as well as the funding and implementation of certain services (such as trauma care) highly political.

Our findings highlight the role played by Syrian providers to access vulnerable populations and their ability to respond to humanitarian norms, as well as their ability to drive change through the system. However, our findings also indicate that the localisation agenda, by capacitating local health workers to assist populations in places where international staff are unable or unwilling to be present, can continue to reproduce the very “hierarchies” it aimed to tackle [29]. The structure of collaborations between international humanitarian actors operating remotely while Syrian actors implementing interventions on the ground raises questions about the principle of “humanity” in relation to what Didier Fassin analyses as the “dialectic between lives to be saved and lives to be risked” [14], and the different values attributed to these lives. In our case study, the question is not only about the price at which humanitarian workers can risk their lives to save lives, but also whose lives should be risked rescuing affected populations. This dialectic is somehow embedded in classic ways of categorising staff between international and local employees within humanitarian aid agencies [14]. Cross-border collaborations between international aid entities working remotely and Syrian health providers who were already delivering aid in the country slowly reshaped these collaborations, especially when Syrian providers were given more space in decision making and when some Syrian NGOs became eligible for direct funding. However, this tension between “lives to be saved and lives to be risked” [14], still needs to be addressed in a context where aid collaborations have institutionalised the practices of Syrian local staff being the only humanitarian workers risking their lives on Syrian ground. In addition, our findings suggest an extension of the politics of life by highlighting how the idea of commitment often translates into blurring the lines between personal, professional and political lives for Syrian health workers who are negotiating the provision of health services under conflict. In this sense, when humanitarian actors arguing for a more cautious implementation of the localisation agenda debate that neutrality might be problematic in a context where humanitarian workers work in a familiar setting, our paper shows the need to engage in supporting local actors to mitigate risks that they (and their families) would be bearing. Furthermore, the security rules that constrain international actors themselves often imply a deliberate and much more limited acceptance of risk, rather than simply external circumstances of the operating context: they too should be considered open to revisit, with an emphasis on equity among people delivering aid, irrespective of their origin. Finally, these reflections on “local actors” and their attempts to build some elements of governance through services’ provision [30], should be accounted for in the reconstruction arrangements ahead, including in areas taken back by the government, where health providers and health institutions could be forcibly reintegrated into the traditional health system of the Ministry of Health. Considering the different potential scenarios in North-West Syria, the experience of local actors so far could contribute largely to rebuilding the health sector in these areas in the future.

## Conclusions

This paper shows that the narrative depicting a humanitarian shift to remote management and localisation from the perspective of international humanitarian organisations lacking access to vulnerable communities inside Syria and deciding to collaborate with local organisations, does not reflect the complex and circular course of this movement. Collaborations between “local” and “international” actors have developed over time through convergent and sometimes competing dynamics, made particularly visible when looking at the delivery of medical aid inside Syria across the Turkish border. The role medical Syrian doctors played early in the conflict in maintaining services and coordinating a health response on the ground, the support provided by diaspora doctors and the international and national efforts developed to allow cross-border humanitarian activities; all participated in progressively institutionalising partnerships between diverse health networks. We argue that there is an opportunity for the global health community to learn from these negotiated convergent dynamics and to rethink how we frame medical humanitarianism (therefore resisting the temptation to mainstream “local” actors into a “traditional” system). Cross-border humanitarian activities facilitate the creation of new rules that are often implicit but that give value to the capacity of the humanitarian system to be flexible and adaptive. Local actors’ legitimacy is built upon their access to populations, and upon their willingness to bear risks that international humanitarian workers would not accept. In this context, “access to populations first” becomes a motto. While learning from the creative and effective bottom-up strategies to enhance cross-border coordination, from the importance of trust and interpersonal relations to develop effective partnerships, and from the emergence of “localisation” as a value, there is a need to reflect on the tensions between international humanitarian principles and to create mechanisms that improve accountability to populations of concern across and beyond “locality” and “proximity to populations”. Learning from these collaborations, the humanitarian and global health communities also need to reflect on how values are attributed to human lives in contexts where national staff risk their lives to save lives in Syria. Finally, this case study highlights the role that professional networks pre-dating the conflict have played in organising, channelling and providing services in areas hard to access by international aid workers. To note, professional networks can include and exclude populations, and their potential to reach vulnerable communities during conflicts should be better understood across sectors.
